# Linking the Resource Description Framework to cheminformatics and proteochemometrics

**DOI:** 10.1186/2041-1480-2-S1-S6

**Published:** 2011-03-07

**Authors:** Egon L Willighagen, Jonathan Alvarsson, Annsofie Andersson, Martin Eklund, Samuel Lampa, Maris Lapins, Ola Spjuth, Jarl ES Wikberg

**Affiliations:** 1 Uppsala University, Department of Pharmaceutical Biosciences, Box 591, SE-751 24 Uppsala, Sweden

## Abstract

**Background:**

Semantic web technologies are finding their way into the life sciences. Ontologies and semantic markup have already been used for more than a decade in molecular sciences, but have not found widespread use yet. The semantic web technology Resource Description Framework (RDF) and related methods show to be sufficiently versatile to change that situation.

**Results:**

The work presented here focuses on linking RDF approaches to existing molecular chemometrics fields, including cheminformatics, QSAR modeling and proteochemometrics. Applications are presented that link RDF technologies to methods from statistics and cheminformatics, including data aggregation, visualization, chemical identification, and property prediction. They demonstrate how this can be done using various existing RDF standards and cheminformatics libraries. For example, we show how IC_50_ and K*_i_* values are modeled for a number of biological targets using data from the ChEMBL database.

**Conclusions:**

We have shown that existing RDF standards can suitably be integrated into existing molecular chemometrics methods. Platforms that unite these technologies, like Bioclipse, makes this even simpler and more transparent. Being able to create and share workflows that integrate data aggregation and analysis (visual and statistical) is beneficial to interoperability and reproducibility. The current work shows that RDF approaches are sufficiently powerful to support molecular chemometrics workflows.

## Background

Molecular chemometrics is the field that finds patterns in molecular information, combining methods from statistics, machine learning, and cheminformatics. We argued before that semantic web technologies are important for lossless exchange of data [1], but it should also be noted that molecular properties are not well described by semantic web technologies alone; similarity of molecular structures is not easily captured by triples, but are required for pattern recognition. Therefore, we will focus in this paper on the interplay between the two kinds of knowledge representation.

Past research in molecular chemometrics has focused mostly on the development and use of statistics and cheminformatics, but semantic technolgies are equally important: the success of the statistical modeling depends very much on the setting up of proper input, as well as the ability to validate the created models against independent information sources afterwards, numerically [2], as well as visually [3]. This requires accurate and meaningful annotation of the data, which reduce the chance of errors introduced by the processes.

This need has been recently met in chemistry by the Chemical Markup Language (CML) [4-6]. CML, however, does not formalize ontologies into the standard, though it does have mechanisms to validate dictionary references. Ontologies have been proposed in chemistry on several occasions too. For example, Gordon used ontologies and reasoning for* chemical inference* in 1988 [7] and more recently, ontologies have been suggested for representation of molecular structures [8-10] and applications in life sciences [11]. Simultaneously, the Open paradigms have been acknowledged as important components to improve cheminformatics [12,13]: Open Data, Open Source, and Open Standards are crucial approaches here, and are promoted by, for example, the Blue Obelisk movement [14].

The Resource Description Framework (RDF) and derived Open Standards, such as the Web Ontology Language (OWL) and the SPARQL query language, are extremely useful technologies. The amount of Open Source software that can use these standards have greatly risen over the past 10 years; these tools now provide the crucial building blocks to handle chemical data expressed in RDF, and include the Jena [15] library and Virtuoso [16] software used in this paper.

What none of these RDF technologies address, however, is the process of converting back and forth numerical and non-numerical representations of knowledge. Neither do they track the errors introduced by these transformations. Converting a drug name into a representation suitable for data mining involves making assumptions, using algorithms introducing computational error, and will reflect errors in databases from which information is retrieved. Likewise, data analysis of a high-dimensional information space is collapsed into a low-dimensional language when we write down arguments and conclusions in publications and submissions to existing databases.

For statistical modeling, validation can address sources of error in translating knowledge in one numerical representation into another. For example, several statistical modeling methods have well-defined equations for the uncertainty of predicted properties. However, at the level of cheminformatics and general knowledge management there is yet no equivalent, but we believe that the Resource Description Framework and related technologies provide us the means to specify these sources of error, so that this information can be used in computation.

It is therefore the topic of this paper to further explore the roles of RDF as a critical component in molecular chemometrics [17]. We show that RDF technologies are sufficiently expressive to allow bridging the gap between non-numerical molecular knowledge representation and numerical representations used in statistical modeling. The next section will first introduce the methods we used to handle and integrate RDF data and how we performed the computations. We will then demonstrate how RDF can be used as input for cheminformatics and statistical studies. We will also show how computation results and computation itself can be done using semantic technologies.

## Methods

We used a number of methods and technologies in this paper to show the possibility for efficiently linking drug discovery related data represented in RDF with cheminformatics and chemometrics. In addition to using existing RDF databases, we made use of newly developed resources using RDF server technologies. The query language SPARQL was used to retrieve data from those servers. The Chemistry Development Kit was used to convert data from the RDF knowledge base into representations suitable for cheminformatics algorithms and statistical analysis, as well as converting the latter back into RDF. The statistical modeling based on RDF data was done in the statistical package R. Visualization of molecular data and integration of other components was done in Bioclipse, and in particular by using the Bioclipse scripting functionality. The following sections discuss these methods in more detail.

### Statistical modeling

Proteochemometric (PCM) models analyze experimentally determined interaction strength of series of ligands with series of proteins. PCM is based on quantitative descriptions derived from structural and/or physicochemical properties of interacting ligands and proteins, which are correlated to interaction affinity using mathematical modeling. PCM models thus reveal molecular properties in ligands and proteins that jointly determine selective interactions. The data set of the PCM model used here as an example comprised interactions of 786 chemical compounds with 8 types of *α*-subunits (i.e. channel pore forming part) of voltage gated sodium channels and 10 types of *α*1-subunits of voltage gated calcium channels, totally 1149 chemical compound-protein pairs. For 162 of these interactions dissociation constant (K*_i_*) values were reported ranging from 0.25 nM to 81 *μ*M. For the remaining 987 interactions IC_50_ values were reported ranging from 1 nM to 100 *μ*M. Although IC_50_ values are dependent on assay conditions and hence are not exactly comparable along multiple assays we here elected to analyze all available data in one PCM model.

We used the Bayesian weighted ridge regression approach [18,19] to predict IC_50_ values against *serine/threonine-protein kinase D3* (ChEMBL target 10885, Swiss-Prot:O94806), from now on referred to as target 10885. Weighted regression was used in order to permit us to take assay confidence into consideration; we gave proportionally higher weights to observations where we had higher confidence in the IC_50_ value obtained from the assay. The Bayesian model can be summarized as:

where **y** is the response variable (i.e. the mean centered logarithm of molecules’ activity scaled to unit variance), **X** the descriptor matrix (mean centered and scaled to unit variance), *β* are the regression coefficients, *σ*^2^ the error variance, *τ*^2^ the variance of the regression coefficients, *n* the number of compound in the dataset retrieved from ChEMBL (i.e. *n* = 449), and *p* is the number of descriptors used (which was 20). Further, **W** and **I** denote the diagonal weight and identity matrices, respectively. The diagonal elements in **W** thus represent the assay confidence for each compound retrieved from the ChEMBL database. To test whether using the assay confidence information improves the predictive ability of the model, we also fitted model (1) using **W***_n_* = **I***_n_* (i.e. equal weight was given to all molecules). The model parameters were estimated using Gibbs sampling as implemented in the JAGS software [20].

### Cheminformatics interpretation

Converting RDF expressed molecular data, such as SMILES strings, into chemical graphs was done using the Chemistry Development Kit (CDK) [21,22]. Calculations of 2D coordinates for diagram visualization in JChemPaint and QSAR descriptors was also performed using the CDK.

### Bioclipse

Bioclipse is a chem- and bioinformatics workbench aimed at integrating local and remote data and computation services [23,24]. It combines integration of these services with visualization capabilities of various life sciences data types, including spectra, molecular structures, and reactions. Bioclipse was used to integrate various applications to provide a unified platform for handling RDF for life sciences related data. Bioclipse was extended to support handling and visualization of RDF data. The Jena RDF library was used to store RDF data as well as read and write data in the RDF/XML and Notation3 [15] formats. The provided triple stores available in Bioclipse include an in-memory store and a Jena-TDB-based on-disk store. Jena also provides an API exposed in Bioclipse to query remote RDF databases using the SPARQL Protocol and RDF Query Language (SPARQL) [25].

For RDF graph visualization in Bioclipse we used the Eclipse Visualization Toolkit Zest [26]. Visualization of 3D molecular structures and 2D molecular diagrams made use of Jmol [27] and JChemPaint [28], respectively. The link between the RDF graph visualization and the JChemPaint molecular editor used the Eclipse extension point mechanism outlined in the Bioclipse papers [23,24].

Bioclipse 2 introduced scripting functionality, and the above outlined functionality is available via managers made available to the Bioclipse scripting environment via Eclipse extension points [23]. Currently, the scripting language available in Bioclipse is JavaScript ("Bioclipse Scripting Language"), and scripts throughout this paper accordingly use this scripting language. The scripts mentioned in this manuscript require a release from the stable Bioclipse 2.4 series or from the matching 2.3 development series. Some of the scripts listed in this paper are available on myExperiment [29].

### RDF graph analysis

Plugins for SWI-Prolog [30] and Pellet [31] have been developed for analysis of RDF graphs. SWI-Prolog provides an environment for running Prolog code and Pellet is an OWL-DL reasoner. Both have been used in this paper for the analysis of RDF graphs. Data stored in files in the Bioclipse workspace is transferred into the Prolog environment with the* swipl.loadRDFToProlog( rdfDataFile )* command. The SWI-Prolog plugin allows to query individual triples with the* rdf_db:rdf( Subject, Predicate, Object )* Prolog command. Wrapper methods have been defined that allow to call this method directly from a Bioclipse script using the* swipl.queryProlog( [”prologMethod”, ”ResultLimit”, ”Param1”, ”Param2”, … ] )* command. Pellet, instead, is integrated into Bioclipse via its Jena interface and can, therefore, operate directly on an existing RDF data store.

### RDF servers

Three RDF servers were set up to provide molecular knowledge in RDF format. The http://rdf.openmolecules.net/ server was set up, using* PHP: Hypertext Preprocessor* (PHP) scripts [32] to dynamically resolve URIs into links to other RDF databases using search facilities of the latter. Two further servers, http://rdf.farmbio.uu.se/chembl/sparql/ and http://rdf.farmbio.uu.se/nmrshiftdb/sparql/, were set up using the Virtuoso 6 software [33] to provide a SPARQL query end points for ChEMBL [34] and NMRShiftDB [35,36] data.

## Results

We here report a number of applications that demonstrate RDF as sufficient technology to integrate knowledge management and databases with cheminformational and statistical analysis. The chosen examples demonstrate the conversion of RDF data into cheminformatical representations and numerical representations for statistical analysis. They also show how cheminformatical representations and computation results can be expressed back into RDF.

### From RDF to cheminformatics

Cheminformatics has provided machine representations of molecular structures for a long time: chemical graphs and derived representations, such as connectivity tables and line notations like SMILES [37] and InChI [38]. Literature, however, defaults to chemical names as labels, which can often only be resolved by means of look-up databases. We present here applications that show how we can use RDF to link non-numerical molecular compound names and labels, to machine readable chemical graph representations.

#### Molecular identity

Retrieval of information about molecular structures from databases is best done with unique identifiers. The InChI has recently acquired a prominent role as unique identifier, and is increasingly used to make resources and literature machine readable [39]. Alternative identifiers, like the Simplified Molecular Input Line Entry System (SMILES), are often not unique, causing relevant data to be lost in the search. 

While it is a unique identifier, the InChI is not ideal for RDF environments: it has a syntax that starts with an* InChI=* prefix, and is not in the URI format used in RDF. To aid the adoption of the InChI in RDF data sets, we have set up a web resource that provides a one-to-one link between the InChI and a URI. Additionally, this URI is dereferenceable, making it suitable for use in LinkedData networks. The dereferenceable nature is important to discover further information, quite like the role of hyperlinks in the world wide web.

**Figure 1:**
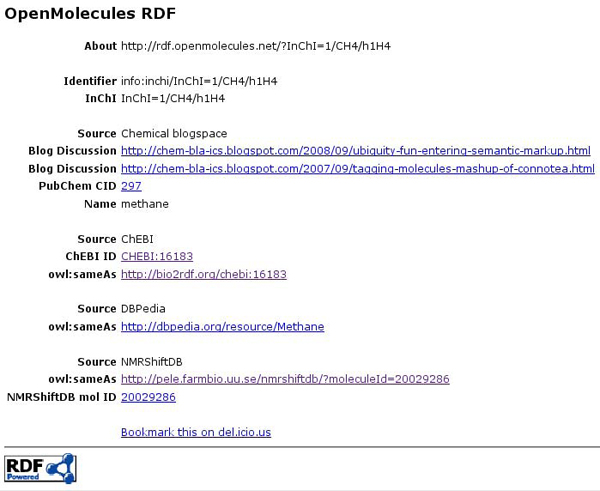
**Screenshot of the *http://rdf.openmolecules.net/* website for methane.** It shows an RDF/XML document visualized by the browser with the associated XSLT stylesheet. Links are made to various resources, showing how the website can serve as hub for linking molecular data using the InChI.

For example, Figure 1 shows the URI-based identifier for methane, http://rdf.openmolecules.net/?InChI=1/CH4/h1H4. The website does not primarily provide new data, but looks up information from other resources and links to those. In this way, it provides autogenerated RDF content for any InChI. These URIs makes it possible for any RDF database to use* owl:sameAs* triples to establish an InChI-based chemical identity for its molecules. Currently, the website acts as a hub in the Linked Data network: links are provided to ChEBI [40], NMRShiftDB [35], and DBPedia [41].

#### Visualization of RDF data

**Figure 2:**
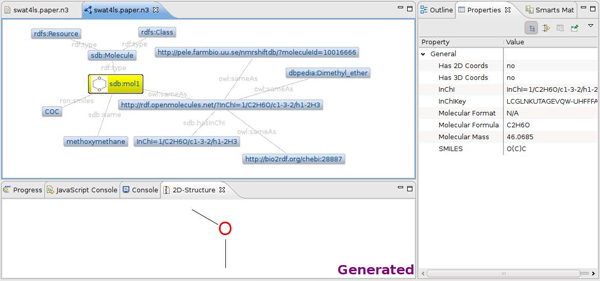
**Screenshot of the visualization in Bioclipse of an RDF graph encoded in a Notation3 file.** The file contains information about methoxymethane (see Figure 3) and links to three further RDF repositories (NMRShiftDB, ChEBI, and DBPedia) connected to via the http://rdf.openmolecules.net/ InChI resolver service. Bioclipse recognized a molecule object with SMILES information, allowing it to compute and visualize further properties, visible by the icon in the RDF graph (yellow node) and the Properties view on the right and the 2D-Structure view down the bottom.

**Figure 3:**
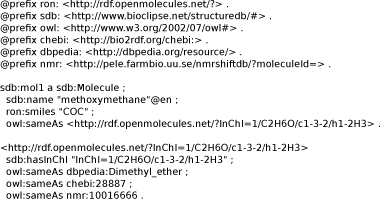
**Notation3 file with a small RDF network for methoxymethane.** Available from additional file 1.

Bioclipse is used in this paper to integrate various RDF functions, and the Zest graph visualization library is used to create a graphical browser for RDF networks. Figure 2 used this functionality and shows a small graph depicting an RDF resource* sdb:mol1,* which is of type* sdb:Molecule* and has a name (*Methanol*) and a SMILES (*CO*). It also has a statement on the molecular identity and a few alternative identifiers from the NMRShiftDB and ChEBI, retrieved via the website http://rdf.openmolecules.net/. This graph visualization functionality in Bioclipse recognizes objects of a supported ontological type, *sdb:Molecule* in the example. The icon in front of the* sdb:mol1* resource indicates that the resource is recognized as a molecule. The icon also implies that Bioclipse knows what to do with such resources. If the user clicks a resource with an icon, it will visualize and compute additional information. Figure 2 shows this in action for the RDF graph shown in Figure 3, where an InChIKey and molecular mass are computed and shown in the Properties view, as well as the matching 2D diagram shown in the 2D-Structure view. Double clicking such a resource will open it in an appropriate Bioclipse editor. For example, this allows a molecule resource in the RDF graph to be opened in a JChemPaint editor.

#### Aggregating data sets

Bioclipse was extended for this paper to provide various ways of aggregating RDF data and now supports two methods to locally cache data: an in-memory data store and an on-disk data store. This makes it possible to download raw RDF data directly into Bioclipse, e.g. using the command

var data = rdf.createInMemoryStore()

followed by

rdf.importURL( data, “http://rdf.openmolecules.net/?InChI=1/CH4/h1H4” ).

Additionally, there is a method to extract RDF from XHTML+RDFa pages [42]:

rdf.importRDFa( data, "http://egonw.github.com/" ).

While these two approaches aggregate RDF data, they do not allow one to query a database. Downloading a full database locally is not often needed, and SPARQL provides a method to query a RDF database for particular bits of information instead. SPARQL queries have been used in earlier discussed visualization applications. It should be stressed that using a common standard like RDF, allows linking of any database; this is not possible when each database uses a distinct format or interface.

The RDF functionality in Bioclipse provides a few new methods to its JavaScript environment:

rdf.sparql( data, someQuery )

and

rdf.sparqlRemote( url, someQuery )

These method allows one to directly query remote SPARQL end points such as those we have set up for the NMRShiftDB and the ChEMBL databases, as discussed in the Methods section. Using this approach we can extract subsets of data from databases. For example, to ask an RDF database for all the predicates, we can run the following command from the Bioclipse JavaScript environment:

var results = rdf.sparqlRemote(

"http://rdf.farmbio.uu.se/chembl/sparql",

"SELECT DISTINCT ?predicate WHERE { [] ?predicate [] }"

)

**Figure 4:**
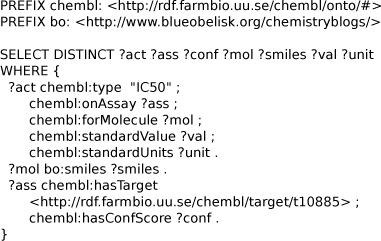
**SPARQL query to extract IC_50_ for target 10885 from ChEMBL.** The query extract information about the assay, binding affinity, an molecular structure (SMILES) of the drug. Available from additional file 2.

**Figure 5:**
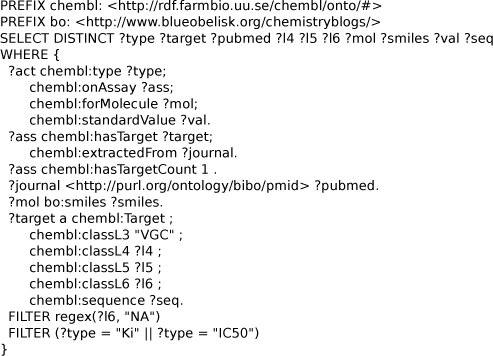
**SPARQL query against the ChEMBL to extract a PCM data set.** The queried data includes IC_50_ and K*_i_* activities against a series of sodium ion channels. Available from additional file 3.

Using this approach, we can focus on the data and the information we want to extract, and rely on the SPARQL standard as reusable, Open API. For example, using this API we can use the SPARQL queries given in Figure 4 and 5 to construct data sets suitable for, respectively, QSAR and proteochemometrics studies.

The SPARQL shown in Figure 4 collects seven related properties from the ChEMBL RDF database, namely: activity identifier (*?act*), assay identifier (*?ass*), molecule identifier (*?mol*), molecular SMILES (*?smiles*), activity value (*?val*) and its unit (*?unit*), and the assay confidence (*?conf*). The query retrieves molecular structures with IC_50_ activities against the 10885 target. The SMILES can, for example, be used by Bioclipse to calculate molecular descriptors for use in statistical modeling. Other retrieved properties can be used in such modeling too, as we will show later.

The SPARQL shown in Figure 5 aggregates even more information, needed for a proteochemometrics study. The query finds molecules that bind targets that are involved in voltage-gated ion channels. The query limits the search results to sodium channels, taking advantage of the ChEMBL classification scheme for targets. The level 6 classification specifies for this target type the ion channel type, and is reflected in the SPARQL query by the *?l6* variable. Only compounds are queried having a K*_i_* or IC_50_ activity. The query retrieves nine properties: target identifier (*?target*) and its type (*?type*), the PubMed ID of the paper from which the activities were extracted (*?pubmed*), three classifications levels (*?l4, ?l5,* and*?l6*), molecule identifier (*?mol*) and the molecule’s SMILES (*?smiles*), activity value (*?val*) and the protein sequence of the targets against which the activities have been measured (*?seq*).

#### Visualization of 2D diagrams and 3D geometries

**Figure 6:**
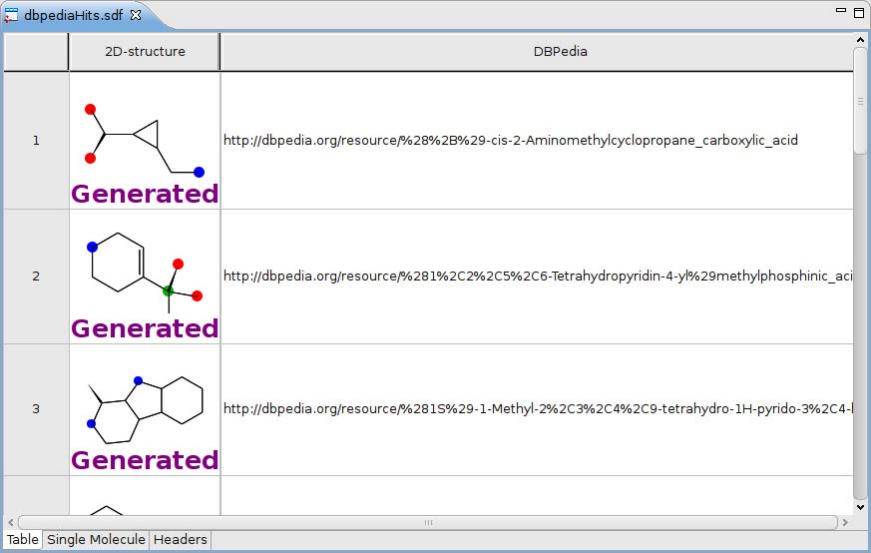
**Screenshot of DBPedia entries with SMILES in Bioclipse.** The data was retrieved with SPARQL and shown in a molecules table by a Bioclipse script (see Figure 7).

We demonstrate the visualization capabilities using a Bioclipse script that queries the SPARQL end point of DBPedia, a RDF database with the structured data from Wikipedia [43]. The script queries all entries that have a SMILES, because those are far more abundant than InChIs in Wikipedia, and it uses the CDK to create an MDL SD file, while storing the DBPedia resource URI as property. Clearly, any chemical property can be calculated on the fly, or looked up via additional RDF sources, as is done in the previous example. The results are then opened in a JChemPaint-based molecule table functionality in Bioclipse, as shown in Figure 6.

**Figure 7:**
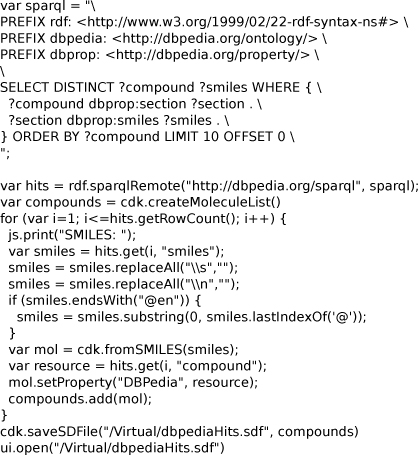
**A Bioclipse script using the DBPedia SPARQL end point to query 10 structures with SMILES and visualizes those.** The found molecules are displayed in a molecule table as is shown in Figure 6. The script is available from MyExperiment.org at http://www.myexperiment.org/workflows/927. Available from additional file 4.

The full Bioclipse script for this application given in Figure 7 shows first a query against the remote SPARQL end point of DBPedia using the* rdf.sparqlRemote( sparql )* call, after which it iterates over all returned hits and extracts the* ?compound* and* ?smiles* fields for each hit as identified in the SPARQL. For each SMILES, the CDK is used to translate the SMILES into a chemical graph which is stored in a list. The list of molecules is finally saved as MDL SD file and opened in a molecules table. 

**Figure 8:**
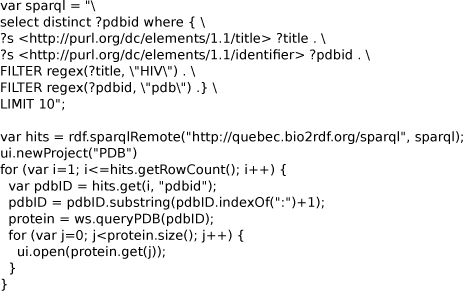
**A Bioclipse script using the Bio2RDF SPARQL end point to query for proteins with ‘HIV’ in their titles.** The found proteins are subsequently opened with the Jmol plugin (see Figure 9). The script is available from MyExperiment.org at http://www.myexperiment.org/workflows/928. Available from additional file 5.

**Figure 9:**
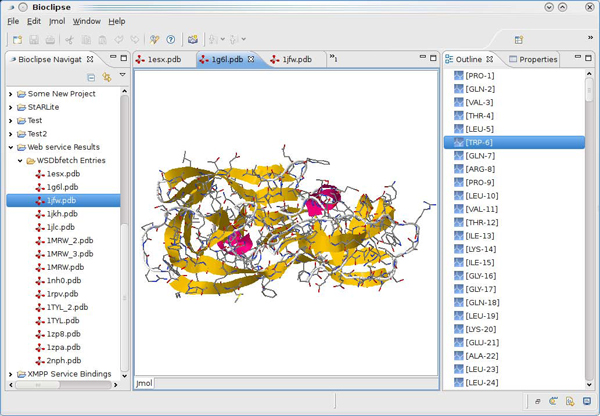
**Screenshot of a Jmol editor in Bioclipse showing a hit for the query against the Bio2RDF SPARQL endpoint for proteins.** The exact query for proteins with the string* HIV* in the title is given in the script in Figure 8.

Bioclipse can also visualize 3D geometries using the plugin for Jmol [27]. The script in Figure 8 uses a SPARQL end point for the Bio2RDF data [44], and looks up protein structures which have a title containing* HIV.* The PDB identifier is extracted and used for a webservice call against the PDB database, and opened in the 3D editor with a* ui.open()* call. Figure 9 shows fifteen downloaded PDB entries in the Bioclipse navigator of which the PDB:1GL6 entry is opened in a Jmol editor. The script is available for download at http://www.myexperiment.org/workflows/928.

### From RDF to chemometrics

The previous sections gave examples of how we can use RDF data in cheminformatics applications. This section shows how to link RDF and statistical analysis field chemometrics. The first example shows how SPARQL is used to retrieve data from RDF sources, and how Bioclipse is used to calculate molecular descriptors to convert the RDF graphs into a numerical representation suitable for statistical analysis. The second and third examples then show how this numerical data is used to find new patterns. The second example shows how to predict IC_50_ values by a Bayesian statistics QSAR study, while the third example additionally takes protein sequences from the ChEMBL database into account, and analyzes the protein-drug interaction in a proteochemometrics setting.

#### Descriptor calculation

Plugins were constructed for Bioclipse to provide convenience methods to access the RDF database with the ChEMBL data at http://rdf.farmbio.uu.se/chembl/. A first plugin provides a Java API for retrieving information from ChEMBL about targets, containing the methods* getProperties(targetID), getActivities(targetID),* and* getQSARData(targetID, activity).* These methods use the SPARQL query functionality of Bioclipse introduced in the previous paragraph, and overcomes the problem of having to construct a full SPARQL query manually. This API is exposed as a Bioclipse manager [23], making these methods available to the JavaScript environment.

**Figure 10:**
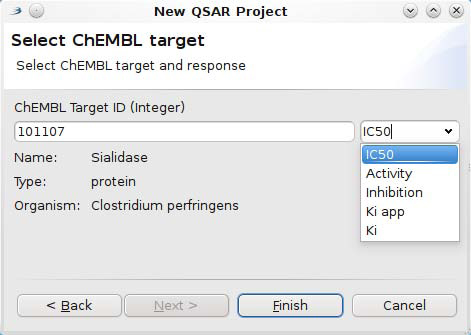
**Screenshot of one of the Bioclipse Wizard pages to set up a new QSAR project.** The wizard allows the user to interactively select a target and activity using SPARQL functionality to download title, type, and organism details for the currently selected target. The wizard automatically updates the list of allowable activity types for the given target, being the sialidase target in this example.

A second plugin uses this new functionality to integrate the ChEMBL SPARQL end point with the QSAR feature of Bioclipse [45]. The plugin provides a* New Wizard* to bootstrap a new QSAR project by aggregating data from the ChEMBL database directly. It accepts a ChEMBL targetID and an activity type (e.g. IC_50_ or K*_d_*), as shown in the screenshot in Figure 10. This new wizard uses SPARQL to update the wizard page with information about the currently given targetID. While the user is typing the targetID number, SPARQL is being used, via the aforementioned wrapping API, to ask the RDF database about the title, type and organism of the current target. Additionally, it will query the database for available activity types, such as the* IC*_50_*, Inhibition, K_i_ app, K_i_*, and a general* Activity* for the 101107 targetID given in the figure. The wizard for Bioclipse does not yet provide full text search for targets based on labels, keywords, and descriptions available in the ChEMBL database, but it is clear that SPARQL make such applications possible too.

When the user is satisfied with the selected target, the* Finish* button can be clicked. The wizard will then download the SMILES and activity values for that target, and serializes all chemical structures into a MDL SD file with the activity scores as properties. Furthermore, it sets up a new QSAR project and populates the project with these structures and responses. The user can then select the descriptors to be calculated for the aggregated molecules and start the computation, all from within Bioclipse.

Thus, the here shown RDF-driven feature makes it straightforward to set up new QSAR datasets for data from the ChEMBL database.

#### IC_50_ modeling

**Figure 11:**
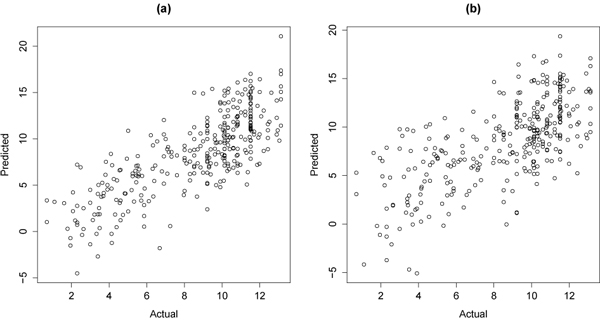
**Actual versus predicted values for a IC_50_ prediction model.** The sub figures show: a) when assay confidence was taken into consideration, and b) when assay confidence was not taken into consideration. The reduced variance of the predicted values suggests that including assay confidence is beneficial to the model’s performance. The prediction model is given in Equation 1.

Using the SPARQL query given in Figure 4 we extracted a QSAR data set from the ChEMBL database. Numerical descriptors were calculated and used as input for the statistical analysis, as described in the previous section. We used a Bayesian weighted ridge regression approach to fit the QSAR model characterizing the relationship between molecular properties of 449 compounds and their extracted IC_50_ activities against the 10885 target. Figure 11 shows the result from a 10-fold cross-validation as actual versus predicted values for model (1) when assay confidence was taken into consideration (Figure 11a) and when assay confidence was not taken into consideration (Figure 11b). It may be noted that including the assay confidence in model (1) seems to improve the predictive performance. The mean predicted residual sum of squares when using the confidence information was 9.3 (7.6; 12.8) compared to 11.2 (8.5; 15.1) when the confidence information was not used (the numbers in parentheses show the 95% Bayesian confidence intervals).

#### Proteochemometric modeling of ion channel inhibition

As a second statistical modeling example, proteochemometric models predicting inhibition were built for ion channel data extracted from ChEMBL by using the SPARQL query given in Figure 5. Properties of chemical compounds were encoded by a set of commonly used molecular descriptors calculated by Dragon Web software, as described [46]. Protein sequences were aligned by ClustalW2, and encoded by physico-chemical property (zz-scale) descriptors of amino acids [47]. To reduce the number of protein descriptors they were subjected to principal component analysis extracting 17 orthogonal variables (principal components). Calculation of ligand-protein cross-terms correlation of descriptors and cross-terms to logarithmically transformed activity data by Partial Least-Squares projections to latent structures (PLS) was performed as described in an earlier paper from our group [46].

**Figure 12:**
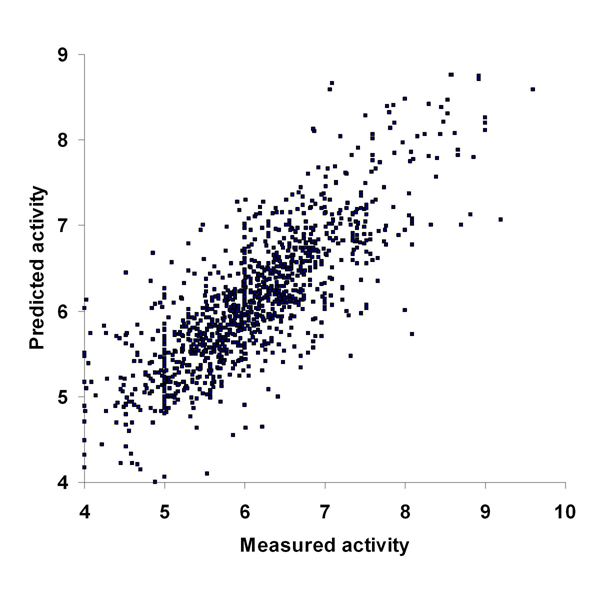
**Correlation of measured interaction activity versus predicted interaction activity.** The correlation is according to a 7-fold cross-validation of the ion channel inhibition model. Activity is expressed as negative logarithm of K*_i_* or IC_50_.

The predictive ability of the induced model was estimated by 7-fold cross-validation, the correlation coefficient between the predicted and experimentally determined values being 0.79 (see Figure 12). The model revealed the most important descriptors for explaining the activity of ion channel inhibitors to be MLOGP (Moriguchi octanol-water partition coefficient), MR (Ghose-Crippen molar refractivity), descriptors of atom centered fragments and functional groups (such as H-046, C-001, C-006, C-033, O-025, O-060, nCaR, nNO2Ph, nNHR, nCrHR; see [48] for explanation of fragment descriptors) and size-related descriptors (molecular weight and mean atomic van der Waals volume). The model also identified molecular properties delineating selective inhibitors of calcium channels from inhibitors of sodium channels.

### From cheminformatics to RDF

In order to fully integrate RDF data with cheminformatics and chemometrics, we need not only to be able to use RDF data as input to algorithms of the latter, but we need also to be able to express cheminformatics knowledge and calculation results from cheminformatics and chemometrics back into RDF. This section shows that RDF is easily able to handle chemical graphs and descriptor calculation output. Also, we demonstrate that traditional cheminformatics algorithms can be rewritten as algorithms directly operating on a corresponding RDF graph.

#### Chemical graphs

The scripts described above were used for QSAR and proteochemometrics, and provided links between protein sequences and drugs. The next integration step is to express data created with cheminformatics as RDF too, and in particular the expression of calculated molecular descriptors as RDF. For this purpose, the data models used by the CDK and the Blue Obelisk Descriptor Ontology (BODO) were expressed as OWL ontologies. The BODO was originally expressed in the Chemical Markup Language [14] by members of the Blue Obelisk movement that promotes Open Data, Open Source, and Open Standards in cheminformatics, and was later translated into OWL by EW. It is used as such in the CDK and in Bioclipse [45]. These ontologies make it possible to express descriptor calculation results as integral part of the Linked Data network.

**Figure 13:**
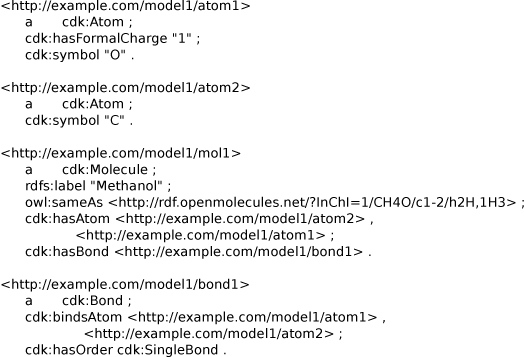
**Notation3 serialization of the CDK data model for protonated methanol.** Methanol is defined as two atoms, one bond in one molecule. A link out to http://rdf.openmolecules.net/ is made using the InChI. Available from additional file 6.

The following example shows protonated methanol as RDF, serialized as Notation3 using the OWL-based CDK data model. It defines a molecule with two atoms, one of which is positively charged. Hydrogens are defined implicitly, as is commonly done in SMILES too. The bond links to the atoms, and has a defined bond order. The resources in the RDF representation match the Java Objects in the CDK library. Java objects are not identified by URIs, which is why the RDF uses* example.com*-based URIs in the example in Figure 13. Alternatively, anonymous resources can be used to reduce the number of URIs, though that puts hierarchical restrictions on how the data is serialized. The current source code that generates the RDF, allows us to use any arbitrary domain, and we anticipate that URIs for all Objects in the CDK will become available when the RDF representation becomes more popular. The Dublin Core namespace is reused for the name of the molecule, and an* owl:sameAs* predicate was used to link to the aforementioned http://rdf.openmolecules.net/ website. The OWL-based CDK data model ontology resembles the actual CDK data model. Compared to a basic chemical graph model, the CDK model has more complexity providing the flexibility needed to cover input from various chemical file formats.

**Figure 14:**
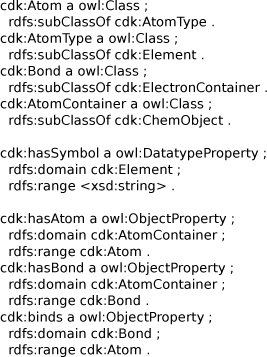
**Subset of the OWL classes and properties describing the CDK data model.** An atom is a subclass of an atom type, which is a subclass of an element; the element has a symbol; an atom container contains atoms and bonds which are subclasses of electron containers; bonds binds two or more atoms.

Besides being able to serialize a CDK model as RDF, the ontology (see Figure 14 for a small subset of the OWL), can also be used to map the CDK data model to other data models at the OWL level. This allows comparing data model ontologies at a more abstract level, possibly even using ontology design tools [8,49]. Reasoning approaches can then be used to determine if the data models are compatible; found incompatibilities highlight potential sources of error when data is translated from one data model to the other. Therefore, the importance of this ontological formulation of the data should be clear.

#### Molecular properties and descriptors

Calculated molecular descriptors can also be added to RDF documents for molecular structures. For this purpose, an extension was written for the above RDF input/output library for the CDK to serialize those descriptors. Serialization of descriptors in a format using semantic web technologies has been proposed earlier to use the Chemical Markup Language [21], and this approach is now extended to directly link to the Blue Obelisk Descriptor Ontology (BODO), as well as to support describing what algorithm parameter values have been used in the descriptor calculation.

**Figure 15:**
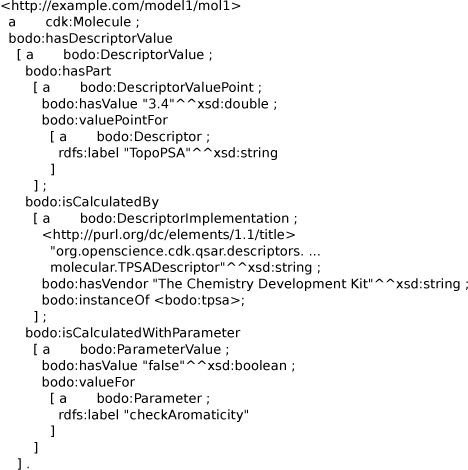
**Notation3 serialization for an RDF graph showing the TPSA descriptor calculation output.** The serialization uses the Blue Obelisk Descriptor Ontology. Besides the actual value, the output also shows how and what calculated the resulting value. Available from additional file 7.

Figure 15 shows the Total Polar Surface Area (TPSA) calculation result for a molecule using the BODO for describing the software, the algorithm, and the parameters the descriptor was calculated with. Shown is that the Chemistry Development Kit was used for the TPSA descriptor, and that the algorithm has one parameter which indicates that aromaticity was not detected before the descriptor was calculated. 

The graph further links to an external dictionary of descriptors that also uses Blue Obelisk Descriptor Ontology; in particular, it refers to the entry describing the TPSA algorithm* (bodo:instanceOf bodo:tpsa*), allowing interoperability as described in the Blue Obelisk paper [14]. The descriptor listing and the underlying ontology are currently found in two OWL documents: one describing the ontology, and the other containing a list of descriptor algorithms [50].

#### Spectral similarity using Prolog

**Figure 16:**
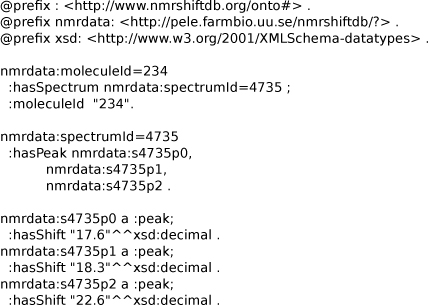
**Notation3 serialization for a RDF graph of a NMR spectrum with three peaks from the NMRShiftDB.** Available from additional file 8.

This last example shows how we can express molecular NMR spectra into RDF and then use reasoning approaches to establish a spectral similarity measure which is otherwise typically done with cheminformatics approaches instead. The example demonstrates how Prolog can be used inside Bioclipse for working with RDF data for the NMRShiftDB. An example RDF representation of an NMR spectrum is given in Figure 16.

Knowledge stored as RDF triples can easily be extended in Prolog by wrapping sets of triples inside Prolog methods with common unbound variables, thereby creating an RDF graph pattern. Using this feature, we can describe larger graph patterns in a uniform way, which is not possible using RDF triples directly. For example, we can combine a set of three RDF triples into a method that expresses the relationship between a molecule and shift values of its associated spectral peaks.

**Figure 17:**
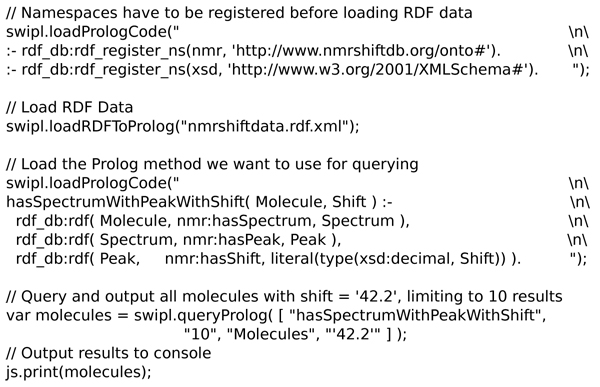
**A Bioclipse script showing the use of the SWI-Prolog functionality to load inline Prolog code.** It uses the loadPrologCode() method, load RDF data with the loadRDFToProlog() method, and query the RDF knowledge base as then defined in the Prolog environment. This particular script searches spectra with a shift near 42.2 ppm. Available from additional file 9.

This approach is used in the script shown in Figure 17 where an RDF file is loaded into the Prolog environment. A Prolog predicate is there defined and then used to query for* molecules which have a spectrum with a peak shift matching the given value.* The resulting molecules are then returned, where they can be opened in a molecules table, if desired, as demonstrated in some of the earlier examples by using the SMILES for the found molecules.

However, we can take things even a step further, taking advantage of the expressiveness of the Prolog programming language by using it directly on the RDF knowledge base. Prolog makes it possible to let one Prolog predicate be composed of sets of other predicates. This makes it it possible to iteratively build upon previously defined* semantics* and thereby step by step increase the expressive power. The Prolog-based code in the* findMolWithPeakValsNear.pl* file provided in this paper’s Additional files section demonstrates this, using more sophisticated code for finding spectra according to a given list of peak shifts that should have near-matches in the database of reference spectra.

**Figure 18:**
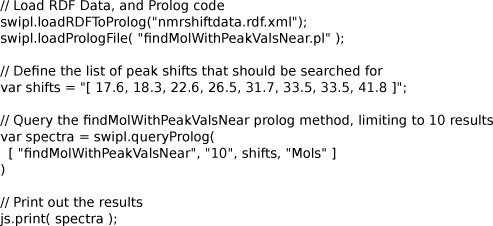
**A Bioclipse script that calls a larger Prolog program to search a spectrum in a database of reference spectra.** This script is available as additional file 10, and the invoked findMolWithPeakValsNear.pl as additional file 11. A similar script is available at http://www.myexperiment.org/workflows/1116.

The code given provides a convenience method to find spectra matching a query spectrum with a number of peaks, as is shown in Figure 18. The Bioclipse script in this figure shows that chemical data expressed in RDF can be used for a typical cheminformatics task, namely the dereplication of a measured NMR spectrum against a database of reference spectra, in this case NMRShiftDB database. The dereplication results are returned to Bioclipse and can be visualized using the spectrum viewer [24].

## Discussion

The applications presented in this paper demonstrate various ways how RDF can be used to represent chemical information and link between data repositories. We also show how SPARQL can be used to query these repositories, and how these emerging standards based on RDF have sufficient expressiveness to cover typical studies in the field of molecular chemometrics. Even though they are sufficient, we can expecte future RDF technologies to enable more elaborate integration.

We must note that the RDF and related standards do not describe how chemical information should be modeled. This leads to a question of which ontologies should be used to markup and annotate the information. This paper uses various ontologies and includes a description of an ontology reflecting the data model used by the cheminformatics library, the Chemistry Development Kit. However, the topic of this paper is not to propose a cheminformatics or a chemistry ontology, but to shows how data expressed in ontologies can be mapped to the implicit ontologies in the various cheminformatics and statistics methods. Aligning with other chemical ontologies, such as ChemAxiom [49] and others [9], is currently being explored.

It is also important to note that RDF and ontologies do not overcome the limitations of what the concepts formalize: while an ontology helps us determine that some string is in fact a SMILES, that knowledge does not overcome the limitations of the SMILES representation as unique identifier. This is why the InChI is preferred, and here used for the http://rdf.openmolecules.net/ project.

Furthermore, we note that expressing similarity between resources only works in a qualitative manner: there are no equivalents of owl:sameAs or rdf:seeAlso that relate two resources given with a certain similarity. It can be argued that such knowledge can be captured with a small set of triples, where the similarity itself is defined as (anonymous) resource, linked to the two similar resources as well as defining the similarity value. However, such information would also require more complex queries to recover and process.

Additionally, neither the SMILES nor InChI contain the information needed by many cheminformatics algorithms: in particular, they do not contain 3D coordinates required by, for example, geometrical descriptors. We believe, however, that RDF contributes here by providing a simple standard that allows linking between databases. By using unique identifiers and the linked data approach, we can take advantage of other available resources that can, independently, contribute additional information, such as 3D molecular geometries.

Our paper does not cover all RDF related technologies, some of which will have a significant impact on the integration of RDF with molecular chemometrics in the future. One of these technologies is XHTML+RDFa [51] which allows embedding RDF data inside common HTML pages. Search engines are already supporting this technology, and we anticipate it to be adopted by the scientific publishing industry too. We predict that in the future data will no longer simply be extracted from dedicated databases, such as ChEMBL, but will be downloaded directly from the scientific literature instead. Such semantic annotation of human-targeted representations, will allow further integration with computing technologies. For example, this embedded RDFa can easily be used by web browser extensions to enrich HTML pages with information from third-party resources [52].

Another technology that will benefit from the integration is the availability of semantic computing services. We have recently started working towards implementing more semantically oriented web services [53], but these do not use RDF technologies yet. Computing services for life sciences that do use RDF standards are being developed by the SADI project [54]. These services allow defining queries that requires calculation of RDF content on demand, providing facilities to look up SADI services that provide information missing from other RDF databases, calling these for the missing information on the fly. This can be used, for example, to make available online descriptor calculation services that output results as RDF.

## Conclusions

This paper shows how RDF data can be integrated with cheminformatics and proteochemometrics using RDF technologies, the CDK and Bioclipse. With these results we argue that semantics in health care and life sciences do not end with giving things names and semantics, but that the domain knowledge includes molecular information, and therefore must involve more technical fields like cheminformatics and proteochemometrics. The use of ontologies in chemistry is not new, but their use in existing tools is minimal. Many current cheminformatics libraries do indeed not have an RDF interface, despite the fact that RDF addresses the important area of data exchange and interoperability. Recent semantic chemistry technologies, like CML, did not use a single open standard, which RDF and its related technologies defines: while CML is restricted to particular chemical data types, RDF is universal, allowing any knowledge base to be linked to the chemical data.

Our examples show how to go back and forth between RDF and a few common cheminformatics representations, including the SMILES, InChI and chemical graphs. They also show how this link can be used to visualize chemical graphs present from online RDF data resources, and how RDF resources can be queried for subsets as input for use in statistical analysis. Further examples highlighted how cheminformatics calculation results can be represented in RDF, and even how traditional cheminformatics methods can be performed directly on RDF graphs using Prolog.

A possible future application of the here presented integration of RDF and molecular chemometrics, is the automatic curation of online (RDF) data repositories. For example, chemical content of Wikipedia can be automatically analyzed for internal consistency as well as consistency with external databases using the RDF version provided by DBPedia. Additionally, missing information can be identified and added. Taking advantage of the scripting functionality in Bioclipse and the sharing of such scripts via myExperiment, such analysis can easily be repeated and used for continuous quality assurance.

We can conclude that existing RDF standards provide the minimal requirements for integrating with existing molecular chemometrics methods. The framework does not solve all problems. For example, accurate and unique identifiers like the InChI are still required to link information sources accurately. The use of platforms like Bioclipse that unite the various technologies makes this simpler and, by using the scripting functionality, more transparent. Being able to create and share the scripted workflows to integrate data aggregation and analysis (both visual and statistical) is beneficial to the field of molecular chemometrics.

## List of abbreviations

BODO: Blue Obelisk Descriptor Ontology; CDK: Chemistry Development Kit; CML: Chemical Markup Language; DL: Descriptive Logic; IC_50_: Half maximal Inhibitory Concentration; InChI: International Chemical Identifier; IUPAC: International Union of Pure and Applied Chemistry; LODD: Linking Open Drug Data; NMR: Nuclear Magnetic Resonance; OWL: Web Ontology Language; PCM: ProteoChemoMetrics; PHP: PHP: Hypertext Preprocessor; PLS: Partial Least-Squares; QSAR: Quantitative Structure-Activity Relationship; RDF: Resource Description Framework; SADI: Semantic Automated Discovery and Integration; SKOS: Simple Knowledge Organization System; SMILES: Simplified Molecular Input Line Entry System; SPARQL: SPARQL Protocol and RDF Query Language; URI: Uniform Resource Identifier

## Competing interests

ME, OS and JW declare financial interest as shareholders in Genetta Soft, a limited Swedish company devoted to software development.

## Authors’ contributions

EW initiated, supervised the project, developed the core RDF functionality in Bioclipse and the CDK, and made the used RDF servers available. ME built the IC_50_ prediction model using the Bayesian statistics. ML built the proteochemometrics model for the ion channel receptor family. OS developed the Bioclipse script to calculate QSAR descriptors. AA developed the SPARQL queries to extract the data used by ME and ML. JA extended the RDF editor functionality for better integration in Bioclipse. SL developed the Prolog plugin which he used in the analysis of NMR spectra. All authors participated in manuscript writing, and read and approved the final manuscript.

## Supplementary Material

Additional file 1 Notation3 file representing methoxymethane. Click here for file

Additional file 2 SPARQL query to extract an IC50 QSAR training data. Click here for file

Additional file 3 SPARQL query to create a proteochemometrics data set for a ion channel proteins. Click here for file

Additional file 4 Bioclipse Scripting Language script that queries DBPedia for small molecules. Click here for file

Additional file 5 Bioclipse Scripting Language script that queries Bio2RDF for HIV proteins. Click here for file

Additional file 6 Notation3 file of methanol using the CDK data model. Click here for file

Additional file 7 Notation3 file showing a QSAR descriptor calculation output. Click here for file

Additional file 8 Notation3 file with an NMR spectrum. Click here for file

Additional file 9 Bioclipse Scripting Language script demonstrating how Prolog code can be run in Bioclipse. Click here for file

Additional file 10 Bioclipse Scripting Language script to search NMR spectra in a database. Click here for file

Additional file 11 Prolog script defining spectral similarity which allows searching the NMRShiftDB RDF data for matching spectra. Click here for file
